# Autonomic Differentiation Map: A Novel Statistical Tool for Interpretation of Heart Rate Variability

**DOI:** 10.3389/fphys.2018.00401

**Published:** 2018-04-23

**Authors:** Daniela Lucini, Nadia Solaro, Massimo Pagani

**Affiliations:** ^1^BIOMETRA Department, University of Milan, Milan, Italy; ^2^Department of Statistics and Quantitative Methods, University of Milano-Bicocca, Milan, Italy

**Keywords:** autonomic nervous system, heart rate variability, spectral analysis, baroreflex, statistics, chronic conditions, prevention

## Abstract

In spite of the large body of evidence suggesting Heart Rate Variability (HRV) alone or combined with blood pressure variability (providing an estimate of baroreflex gain) as a useful technique to assess the autonomic regulation of the cardiovascular system, there is still an ongoing debate about methodology, interpretation, and clinical applications. In the present investigation, we hypothesize that non-parametric and multivariate exploratory statistical manipulation of HRV data could provide a novel informational tool useful to differentiate normal controls from clinical groups, such as athletes, or subjects affected by obesity, hypertension, or stress. With a data-driven protocol in 1,352 ambulant subjects, we compute HRV and baroreflex indices from short-term data series as proxies of autonomic (ANS) regulation. We apply a three-step statistical procedure, by first removing age and gender effects. Subsequently, by factor analysis, we extract four ANS latent domains that detain the large majority of information (86.94%), subdivided in oscillatory (40.84%), amplitude (18.04%), pressure (16.48%), and pulse domains (11.58%). Finally, we test the overall capacity to differentiate clinical groups vs. control. To give more practical value and improve readability, statistical results concerning individual discriminant ANS proxies and ANS differentiation profiles are displayed through peculiar graphical tools, i.e., significance diagram and ANS differentiation map, respectively. This approach, which simultaneously uses all available information about the system, shows what domains make up the difference in ANS discrimination. e.g., athletes differ from controls in all domains, but with a graded strength: maximal in the (normalized) oscillatory and in the pulse domains, slightly less in the pressure domain and minimal in the amplitude domain. The application of multiple (non-parametric and exploratory) statistical and graphical tools to ANS proxies defines differentiation profiles that could provide a better understanding of autonomic differences between clinical groups and controls. ANS differentiation map permits to rapidly and simply synthesize the possible difference between clinical groups and controls, evidencing the ANS latent domains that have at least a medium strength of discrimination, while the significance diagram permits to identify the single ANS proxies inside each ANS latent domain that resulted in significant comparisons according to statistical tests.

## Introduction

The burden of chronic conditions, such as obesity (Christakis and Fowler, 2007) or hypertension (Forouzanfar et al., 2017), is continuously growing worldwide and represents an important barrier to modernization in developing countries. Lifestyle optimization (Ding et al., 2015), focusing on better nutrition, more active life, and management of stress, represents a potentially useful intervention strategy. Advantages may be obtained both organizationally and economically. The extent of the problem and the emergence of new large-scale technologies suggest that novel approaches and types of analysis might provide a fresher point of view to seemingly established conditions, such as obesity or physical activity (Althoff et al., 2017). Lifestyle therapy aims at combating obesity, increasing physical activity, and reducing stress, potentially improving autonomic cardiac regulation. Notably, autonomic regulation can be assessed non-invasively by computer analysis of beat by beat RR interval (more frequently indicated as Heart Rate Variability, HRV) (Task Force of the European Society of Cardiology and the North American Society of Pacing and Electrophysiology, 1996) and arterial pressure variability.

The success of clinical applications heavily depends on the balance between complexity of technique and ease of use (Abraham and Michie, 2008). Thus it should not surprise that HRV (Task Force of the European Society of Cardiology and the North American Society of Pacing and Electrophysiology, 1996) alone gained a broad interest for clinical applications. However, there are several methodological criticalities:

- with protocols (frequently based on laboratory conditions, and relatively small samples), and
- with methods of analysis and interpretation (Billman et al., 2015; Sassi et al., 2015).
  Regarding this latter one, we should consider:
- the length of data series (long-term, typically 24 h, or short-term, usually 5–10 min),
- the techniques employed to extract autonomic indices (time or frequency domain, deterministic, or pseudo-stochastic),
- the interpretative codes (Gerstner et al., 1997) of underlying activity and syntax (Buzsáki and Watson, 2012) of autonomic neurons (amplitude, oscillations, coherence, phase, etc.) and of multiple indices that are provided by the analysis of HRV.

For instance, it is well-recognized that to interpret neural activity we must consider a large set of coding modalities. Conversely, the majority of studies on RR interval or its variability give less relevance to the embedded codes of higher order (Pagani and Malliani, 2000).

The usual focus is on the different value of raw and normalized units in assessing the interaction between low- and high-frequency components (LF and HF) of HRV as a proxy of the neural balance between sympathetic and vagal modulation (taken as indices of excitatory/inhibitory influences; Pagani et al., 1986). This latter view is in line with historical models (Hess, 2014) and with electrophysiological studies with single unit recordings of efferent vagal fibers (Schwartz et al., 1973). Overall these studies support a dual antagonistic sympathetic/parasympathetic innervation of SA node. A definite improvement in the strength of clinical prediction, particularly in cardiac conditions, is offered by the addition of the cardiac baroreflex (La Rovere et al., 1998), assessed either in time (baroreflex slope) (Bertinieri et al., 1985) or frequency domain (index alpha) (Pagani et al., 1988).

A more-in-depth understanding of the hidden meaning of various autonomic proxies could be achieved using specific statistical tools. By Principal Component Analysis (Tarvainen et al., 2014) or Factor Analysis (Fukusaki et al., 2000), one can focus on the less explored hypothesis that information distributed across HRV derived variables could be exploited simultaneously, or again, by discriminant analysis (Jeong and Finkelstein, 2015), used for a more efficient separation in clinical groups. In particular, latent factor statistical methods may also help identify homogeneous clusters of few variables capable of exploring the pathophysiology underlying HRV characteristics. For instance, in relatively large groups of participants, mathematical forecasting showed that the major part of information (>80%) predicting the stand induced sympathetic excitation in normal humans is concentrated in only three variables (RR interval, LF and HF in nu; Malliani et al., 1997). Moreover, a logistic regression modeling approach showed that the autonomic information predicting the hypertensive state is concentrated on RR variance, the stand induced increase in LF nu, and the index alpha (Lucini et al., 2014).

Following this rationale, we hypothesize that a data-driven, pragmatic study protocol (Ford and Norrie, 2016), using multiple statistical methods in an integrated way for detecting latent domains (Thompson, 2004), could provide a novel approach to assess which autonomic (ANS) clusters might define profiles with the greatest discriminant capability across different clinical conditions. Specifically, we start from the assumption that groups such as athletes, normal subjects, obese subjects, people with high stress and hypertensives, overall form a physiological-pathological continuum of ANS regulation and dysregulation that could be captured by statistical tools that are not model-based. This is a crucial point. Setting up a statistical model requires specifying a functional form plus a set of conjectures about the data distribution through which a dependent variable, e.g., the probability of membership to a group, is linked to a set of good explicative variables, as, e.g., in the multinomial logistic model. Statistical modeling could, however, carry with it that data be severely forced within a too stringent statistical-mathematical formulation, which could even lead to poor fitting. We argued that this is particularly true in the context of ANS variables (or proxies), where relationships among them are still substantially under investigation (e.g., the difference between the raw and normalized power of LF and HF oscillations; Pagani et al., 1986). Moreover, most ANS proxies are typically not normally distributed, so that application of classical methods of statistical inference could lead to misleading conclusions. Also, it would be useful to provide practical indications about which ANS proxies could help distinguish subjects outside a “normal” ANS condition. In this sense, we treat subjects without pathologies (normal group) as the reference condition and compare, with respect to this, the other states along the ANS continuum (Narkiewicz and Somers, 1998).

All these considerations influenced the choice of the statistical approach where the primary concern was avoiding potential bias in the analyses. We then preferred to rely on non-parametric and multivariate exploratory statistical techniques and use them in an integrated manner rather than refer to statistical modeling (e.g., multinomial logistic regression model) or discriminant analysis (e.g., linear or quadratic discriminant analysis, which requires multivariate normality of data; Jobson, 2012). More specifically, we assess on a relatively large population of ambulant subjects with an expected wide variation of autonomic performance (from good to poor) whether clinical (or test) groups (athletes, obese subjects, people with high stress, and hypertensives) can be differentiated from controls (normal group) according to differences in ANS latent domains. ANS latent domains that prove to be capable of distinguishing clinical groups from controls are the constitutive elements of what we regard as ANS differentiation profiles of the clinical groups. We set up such profiles in a three-step analysis, the first of which is the preliminary handling of the ANS proxies. Since these latter ones are affected by age and gender effects, we first compute adjusted (Adj) ANS proxies, which are free from such effects, and use them throughout the analyses to detect the ANS differentiation profiles for each test group. We use non-parametric statistical procedures (Bowman and Azzalini, 1997; Hollander et al., 2014) to disclose individual Adj-ANS proxies that are capable of recognizing differences between test and normal groups. Then, we employ factor analysis to reduce the ANS proxies into few ANS latent domains (Thompson, 2004) and assess their overall capacity to recognize clinical groups vs. controls by exploiting the results achieved for the individual discriminant ANS proxies. Lastly, to give more practical value and improve readability, statistical results concerning individual discriminant ANS proxies and ANS differentiation profiles are displayed through peculiar graphical tools, i.e., significance diagram and ANS differentiation map, respectively. Implicitly this approach supports novel hypotheses between statistical properties of data clusters and underlying physiological organization (Pagani and Malliani, 2000).

Because of the complexity of the statistical approach and richness of the results, a large part of them is omitted from the text and presented in the Supplementary Material. We will not however make specific reference to it throughout the text.

## Methods

Data for this study, which is part of an ongoing series of investigations, focused on the use of autonomic indices in cardiovascular prevention. They refer to a population of 1,352 ambulant subjects, who visited our outpatient Exercise Medicine Clinic for reasons varying from a health check-up to cardiovascular prevention (Lucini and Pagani, 2012) for obesity, stress, or hypertension, or the annual pre-participation sport screening (see Table 1). Data were excluded from the study if subjects were outside the range of 18–75 years, if they were smokers (any quantity), or affected by acute diseases (within 3 months), or treated with drugs known to interfere with autonomic cardiovascular regulation or performance. The protocol of the study followed the principles of the Declaration of Helsinki and Title 45, US Code of Federal Regulations, Part 46, Protection of Human Subjects, Revised 13 November 2001, effective 13 December 2001 and was approved by the Independent Ethics Committee of IRCCS Humanitas Clinical Institute (Rozzano, IT). All subjects gave their informed consent to participate.

**Table 1:** Frequency and percentage distributions of participants within clinical groups.

**Groups**	**Count**	**Percentage**	**Description**
Athlete	149	11.0%	Competitive sports, e.g., basket players, football players, badminton players, cyclists, rowers: Years of intense training and participation to competitions
Normal	547	40.5%	Non-smoking subjects without pathologies
Obese	102	7.5%	Subjects with BMI ≥ 30 (kg/m^2^)
Stress	190	14.1%	Psychological dimension of stress: Presence/absence of stress according to self-report of participants who asked advice for stress symptoms lasting more than three months, or referral by their physicians
Hypertensive (HT)	271	20.0%	Subjects with Systolic BP ≥ 140 mmHg or Diastolic BP ≥ 90 mmHg, or both
HT-Obese	55	4.1%	Obese subjects with high BP
HT-Stress	38	2.8%	Stressed subjects with high BP
**Total**	1352	100.0%	

### Autonomic evaluation

The day of recordings, all individuals arrived at the laboratory at least 2 h after a light breakfast, avoiding caffeinated beverages and heavy physical exercise in the previous 24 h. To account for circadian variations, acquisition of ECG (single thoracic lead) and respiration (piezoelectric belt) (Marazza, Monza, Italy), and arterial pressure waveforms (Finapres, TNO, Netherlands), were always performed between 10.00 and 12.00 h. Following our usual procedure, continuous signal (ECG, respiration, and arterial pressure waveform) acquisition was obtained for at least 5–7 min at rest and 5 min upon standing up. As described previously (Pagani et al., 1986), from the autoregressive spectral analysis of RR interval and systolic arterial pressure (SAP) variability, a series of indices indirectly reflecting cardiovascular autonomic modulation was derived, with minimal operator involvement thanks to a dedicated software (Badilini et al., 2005; see Table 2).

**Table 2:** Definition of the variables (ANS proxies) employed in the study.

**Vars**.	**Units**	**Definition**
HR	beat/min	Heart Rate
RR Mean	msec	Average of RR interval from tachogram
RR TP	msec^2^	RR variance from tachogram
RR LFa	msec^2^	Absolute power(a) of Low Frequency (LF) component of RR variability (V)
RR HFa	msec^2^	Absolute power(a) of High Frequency (HF) component of RRV
RR LFnu	nu	Normalized power(nu) of Low Frequency (LF) component of RRV
RR HFnu	nu	Normalized power(nu) of High Frequency (HF) component of RRV
RR LF/HF	–	Ratio between absolute values of LF and HF
RR LFHz	Hz	Center frequency of LF
RR HFHz	Hz	Center frequency of HF, providing a measure of respiratory rate
ΔRRLFnu	nu	Difference in LF power in nu between stand and rest
α index	msec/mmHg	Frequency domain measure of baroreflex gain
SAP	mmHg	Systolic arterial pressure by sphygmomanometer
DAP	mmHg	Diastolic arterial pressure by sphygmomanometer
SAP Mean	mmHg	Average of systogram (i.e., systolic arterial pressure variability by Finometer)
SAP LFa	mmHg^2^	Absolute power of LF component of systogram

We use (Pagani et al., 1986) an autoregressive algorithm to automatically compute power and frequency of spectral components in the bandwidth of interest, discarding components of <5% power that are treated as noise. The software tool (Badilini et al., 2005) is set as to consider components with a center frequency of 0.03–0.14 Hz as Low Frequency, and components within the range 0.15–0.35 Hz as High Frequency, recalling that “the HF component is synchronous with the respiration” (Pagani et al., 1986), using a high coherence between RR variability and respiration as a confirmation. Recordings of subjects with low-frequency breathing are discarded to avoid entrainment, and biased increased LF power (Lucini et al., 2017).

The sensitivity of arterial baroreflex control of RR interval was also assessed by a frequency domain method (α index = average of the square root of the ratio between RR interval and SA Pressure Spectral powers of the low-frequency and high-frequency components; Pagani et al., 1988). In all individuals included in the study, respiratory rate coincided with the high-frequency component of RR variability.

### Statistics

Individuals were divided into 7 clinical groups, from athletes to hypertensive-stressed subjects (see Table 1). The majority of individuals (40.5% out of 1,352) fell into the normal group, which was regarded as the reference group. The other groups were treated as test groups to be compared with the normal one. Groups were chosen according to the likelihood of presenting a condition of putative higher vagal drive, as expected in elite athletes at midseason (Iellamo et al., 2002), or of excessive sympathetic drive, as expected in patients (Mancia and Grassi, 2014) with obesity, hypertension or stress (Lucini and Pagani, 2012). That is in line with the study hypothesis that various conditions might show different (possibly specific) differentiation profiles of autonomic (ANS) proxies (Table 1). To account for intertwined clinical conditions and carry out statistical analyses *ceteris paribus*, subjects who presented a concurrence of obesity and hypertension, or stress and hypertension, were aggregated into two groups: HT-Obese (4.1%, Table 1) and HT-Stress (2.8%, Table 1). On the other hand, subjects having either stress, and obesity together, or stress, obesity, and hypertension together, were discarded from the study because of their too exiguous number (5 and 2 subjects only, respectively). Apart from these situations, no other form of concurrence of different status was observed.

**Figure 1:**
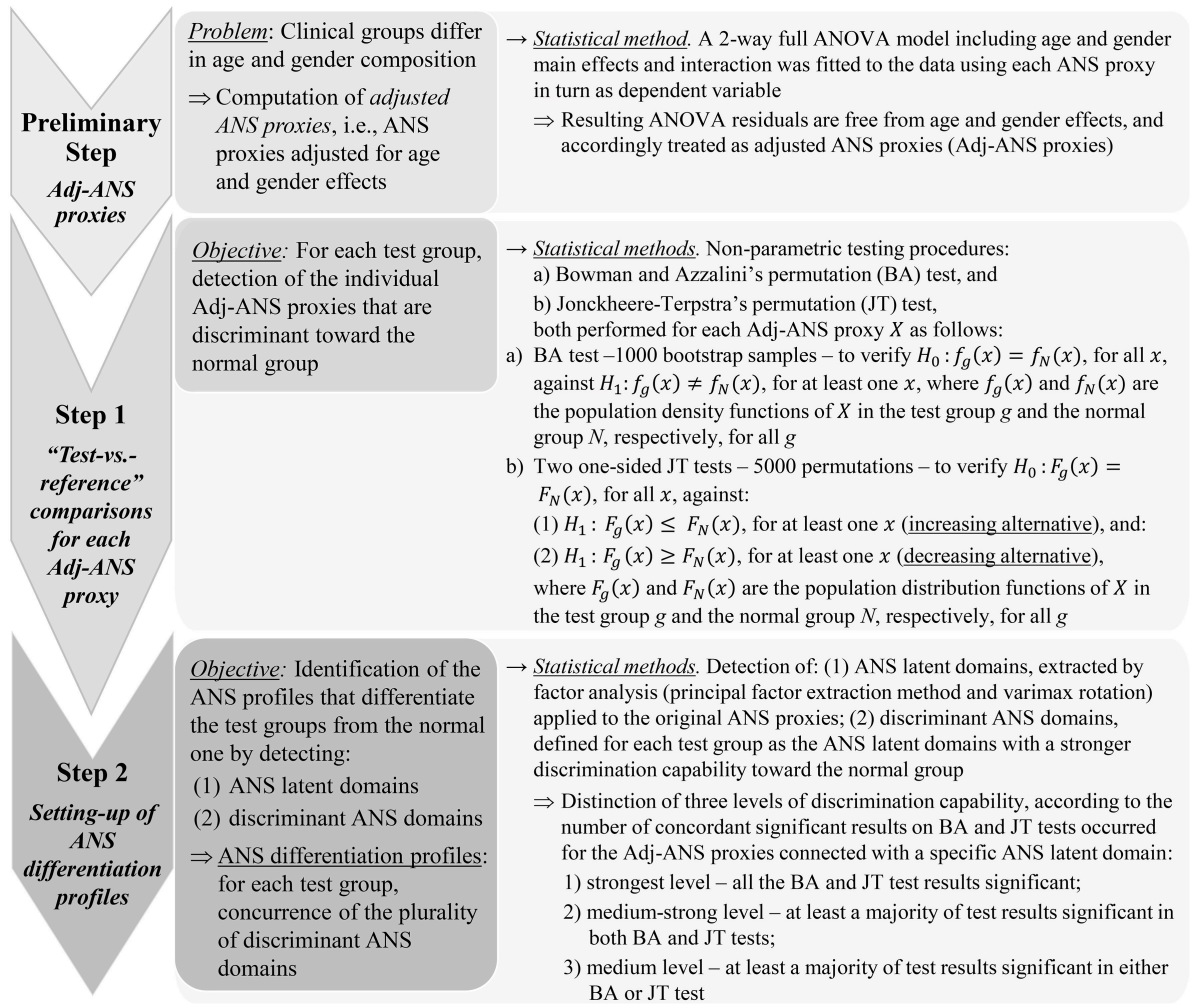
Statistical analysis steps for the detection of ANS differentiation profiles.

The main aim of the study was the detection of ANS profiles capable of distinguishing each test group from the normal one in a “real life” ambulant population (Ford and Norrie, 2016). We refer to these profiles as *ANS differentiation profiles*. Figure 1 sums up statistical analysis steps carried out for their detection. A crucial issue affected the choice of the statistical approach. Clinical groups were not directly comparable because of their different composition in terms of age and gender. For instance, 71.1% of athletes were male, and 75.6% of obese individuals were female; 98% of athletes were under 34 years of age, while 48% of hypertensive subjects were over 50 years. Setting up ANS differentiation profiles by working within “age-by-gender” classes did not prove to be a convenient solution in this case. This choice would have meant dealing with empty subgroups or subgroups too small in size (e.g., there was no female athlete over 50 years of age). Comparability among the groups was thus attained statistically by removing age and gender effects from the considered ANS proxies. A 2-way full ANOVA model including age and gender main effects plus their interaction was fitted to each ANS proxy, and ANOVA residuals, being free of such effects, were used as so-called *adjusted ANS proxies* (Adj-ANS proxies; Figure 1, preliminary step).

ANS differentiation profiles were thus built using the Adj-ANS proxies, instead of the original ANS proxies, through the further two-step analysis outlined in Figure 1 (steps 1 and 2). The aim was first to detect single ANS proxies capable of distinguishing the test groups from the normal one (step 1), and subsequently, from this knowledge, set up discriminant ANS domains in order to reduce the overlapping information of the ANS proxies to a small number of latent dimensions (step 2). Specifically, in the first step, each within-test-group distribution of the Adj-ANS proxies was compared to the corresponding within-normal-group distribution through the non-parametric testing procedures by (a) Bowman and Azzalini's permutation (BA) test (Bowman and Azzalini, 1997), and (b) Jonckheere-Terpstra's (JT) permutation test (Hollander et al., 2014; Seshan, 2017). The nominal significance level was set at 0.05. Regarding the BA test, rejection of the null hypothesis for a specific ANS proxy in a “test-vs.-reference” comparison indicates, controlling for age and gender effects, that the ANS proxy distribution for that test group in the population has a shape generically different to the normal population. By the permutation JT test, the nature of these shape differences was investigated further. For every “test-vs.-reference” comparison involving each Adj-ANS proxy, the null hypothesis of equality between distributions was tested using two separate one-sided JT tests: the first against the so-called increasing alternative, the second against the decreasing alternative (Figure 1, step 1). Rejection of the null hypothesis in favor of an increasing (decreasing) alternative would evidence, net of age and gender effects, that the ANS proxy distribution is more highly concentrated around smaller (higher) values in the test rather than the normal population.

In the second step (Figure 1), ANS differentiation profiles were set up for each test group in the light of the results achieved separately for the Adj-ANS proxies in step 1. This was accomplished by:

- detecting latent domains underlying the observed ANS proxies (i.e., *ANS latent domains*), to obtain a limited set of unobserved, uncorrelated dimensions of the ANS system that are practically measured by the plurality of the ANS proxies. This was carried out through factor analysis (Thompson, 2004; principal factor extraction method with varimax rotation) applied to the original ANS proxies. We regard the first common factors each explaining a substantial percentage (i.e., at least 10%) of the total communality (i.e., the part of total variance reproducible by common factors) as ANS latent domains. And then:
- identifying, for each test group, the ANS latent domains of stronger differentiation capability against the normal group (according to the definition reported in Figure 1, step 2) using the BA and JT test results of step 1. We refer to such domains as *discriminant ANS domains*.

Regarding point (ii) above, discrimination capability of an ANS latent domain was appraised for each test group by the number of jointly significant results on BA and JT tests that occurred for the Adj-ANS proxies connected with that specific domain. In this regard, it is worth remarking that BA and JT tests might not lead in general to concordant inferential results because these procedures are based on far different theoretical grounds, and therefore they can capture different aspects in the comparison between two statistical distributions. Nonetheless, a significant BA test followed by a not significant JT test could reveal that two distributions differ in shape, but not in position, because of a major/minor concentration of points in the central part or in the tails of one of the two distributions. The opposite situation (a significant JT test and a not significant BA test) would indeed be less clearly interpretable. From a practical point of view, these situations could indicate weak empirical support toward the alternative hypothesis of difference between distributions.

Statistical analysis ended with the set-up of ANS differentiation profiles for each test group against the normal group. These profiles were given for each test group by the concurrence of the plurality of discriminant ANS domains detected according to the above procedure. To provide a more immediate clinical value, ANS differentiation profiles were visualized through a graphical map (ANS differentiation map) containing different color grades according to the strength of their discrimination capability.

**Figure 2:**
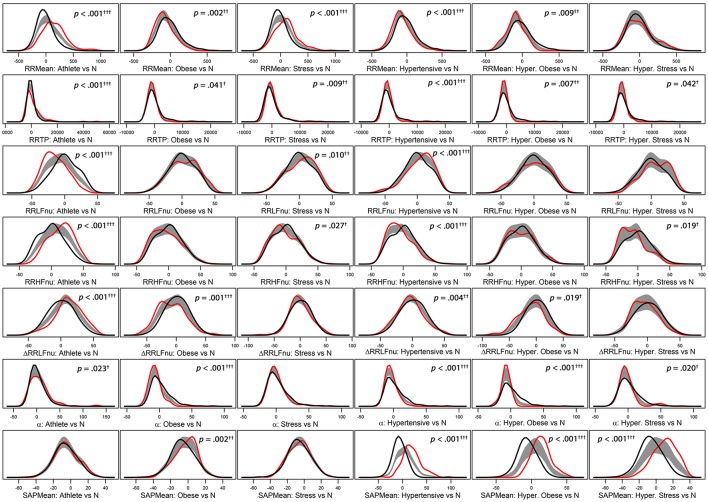
Panel plot of estimated density curves* of six selected adjusted ANS proxies (i.e., RR Mean, RR TP, RR LFnu, RR HFnu, ΔRRLFnu, α index, and SAP Mean). Gray regions represent reference bands for equality between the curves of each test group against the normal group (N).*Density estimates are obtained through the kernel density method by Bowman and Azzalini (1997). Roughly speaking, histogram of each adjusted ANS proxy is interpolated in order to have a smoothed empirical density curve. Colors in each panel: the black curve refers to the normal group (reference group); the red curve concerns a specific test group (Athlete, Obese, Stress, Hypertensive, HT-Obese, or HT-Stress, resp.) regarded for the comparison with the normal group. The gray region is a reference band for equality between the two curves. Panels with significant results include *p*-values and significance codes: 0.001^*†††*^, 0.01^*††*^, 0.05^*†*^.

BA test, along with the smoothed density curves appearing in Figure 2, and JT permutation test were carried out with software R ver. 3.4.0 (R Core Team, Vienna, Austria, 2017) and the libraries “clinfun” (Seshan, 2017) and “sm” (Bowman and Azzalini, 2014), respectively. Descriptive statistics, construction of Adj-ANS proxies through the 2-way full ANOVA model and factor analysis were carried out with software SAS ver. 9.4.

## Results

Total and within-groups mean and standard deviation of the considered 16 ANS proxies (definitions in Table 2) are presented in Table 3.

**Table 3:** ANS proxies: Descriptive data (mean and standard deviation) within clinical groups and over the groups.

	**Groups**															
**Vars**	**Athlete**		**Normal**		**Obese**		**Stress**		**Hypertensive**		**HT-obese**		**HT-stress**		**Total**	
	**Mean**	***SD***	**Mean**	***SD***	**Mean**	***SD***	**Mean**	***SD***	**Mean**	***SD***	**Mean**	***SD***	**Mean**	***SD***	**Mean**	***SD***
HR	55.54	11.22	67.02	10.39	73.85	11.05	61.27	12.40	71.83	11.39	73.82	10.66	66.39	12.01	66.69	12.31
RR Mean	1124.00	227.53	917.62	148.68	831.11	128.45	1019.18	206.29	855.47	131.71	828.11	110.57	934.15	176.69	932.48	184.94
RR TP	6086.07	6843.65	3184.62	3414.38	1643.55	1689.91	2616.41	2388.96	1507.40	1412.89	1068.84	1189.08	1718.37	1266.80	2844.80	3632.93
RR LFa	1180.54	1186.36	1022.47	1491.71	501.45	608.27	804.60	1006.64	460.29	530.03	355.92	421.25	556.36	815.79	818.26	1170.04
RR HFa	2672.21	3800.57	998.40	1672.46	378.76	511.09	664.46	999.15	294.30	594.16	222.18	357.88	231.21	280.84	905.92	1878.27
RR LFnu	34.98	17.92	51.16	20.33	53.92	21.94	54.47	22.19	58.59	20.49	54.69	22.95	61.28	20.95	51.97	21.65
RR HFnu	59.53	19.08	41.61	20.23	37.51	21.36	37.74	21.61	31.75	18.73	36.57	20.91	29.66	18.92	40.21	21.53
RR LF/HF	0.82	0.91	2.28	3.24	4.29	9.87	3.34	4.97	3.99	5.82	3.18	4.59	5.98	12.33	2.90	5.31
RR LFHz	0.10	0.02	0.10	0.02	0.09	0.03	0.09	0.02	0.10	0.03	0.09	0.03	0.09	0.02	0.10	0.03
RR HFHz	0.27	0.06	0.27	0.06	0.30	0.07	0.25	0.06	0.28	0.06	0.30	0.08	0.23	0.06	0.27	0.06
ΔRRLFnu	47.57	20.92	27.44	20.54	14.45	23.40	23.05	21.90	15.44	20.21	7.95	23.12	15.85	17.76	24.55	23.20
α index	34.78	22.62	24.67	16.25	12.99	9.24	20.41	14.81	11.45	8.31	8.11	4.06	15.45	14.72	20.42	16.26
SAP	112.53	15.36	113.55	11.86	119.49	9.88	117.26	12.63	147.75	16.67	149.47	15.01	140.00	11.45	123.62	19.90
DAP	68.63	7.62	70.87	7.71	75.11	6.80	74.26	8.46	93.50	9.62	93.76	11.61	91.66	5.71	77.58	12.87
SAP Mean	108.62	12.91	114.38	12.49	120.30	12.06	119.17	13.69	150.95	19.91	144.06	12.26	140.34	14.41	123.50	20.19
SAP LFa	4.28	4.46	4.12	5.79	4.96	6.75	4.82	6.30	5.70	8.50	6.61	8.25	6.23	8.91	4.78	6.66

Regarding the “test-vs.-reference” comparisons analysis (step 1, Figure 1), Figure 2 provides a graphical representation of the BA test, which addresses the logic behind it. Empirical density functions of a variable, rather than a usual summary measure (e.g., the mean), are compared in their entirety across two different groups and without assuming a priori hypotheses on the data distribution. In such a way, the overall shape of the distribution, and not only a single value, is considered for comparisons. Pointwise comparisons are expressed by means of a non-parametric 95%-confidence region (“reference band for equality”). If two curves lie both inside it, then they are accepted as equal. Otherwise, they are significantly different. Specifically, in Figure 2 estimated density curves of the distribution of six selected Adj-ANS proxies in the test groups (red curve) and the normal group (black curve) are depicted, and the reference band for equality (gray region) is juxtaposed to show pointwise equality/difference between the curves (Bowman and Azzalini, 1997). Overall, it is apparent that different clinical conditions translate into diverse profiles of autonomic differentiation from normal group. For instance, the last row of panels in Figure 2 shows that, after controlling for age and gender effects, the distribution of SAP Mean is different from the normal group in all but athletes (first panel) and stressed individuals (third panel). In these latter two groups, the reference band exactly contains both the red and the black curves (i.e., equality of the curves), while this does not occur for the other groups (i.e., notice the difference between the curves).

**Figure 3:**
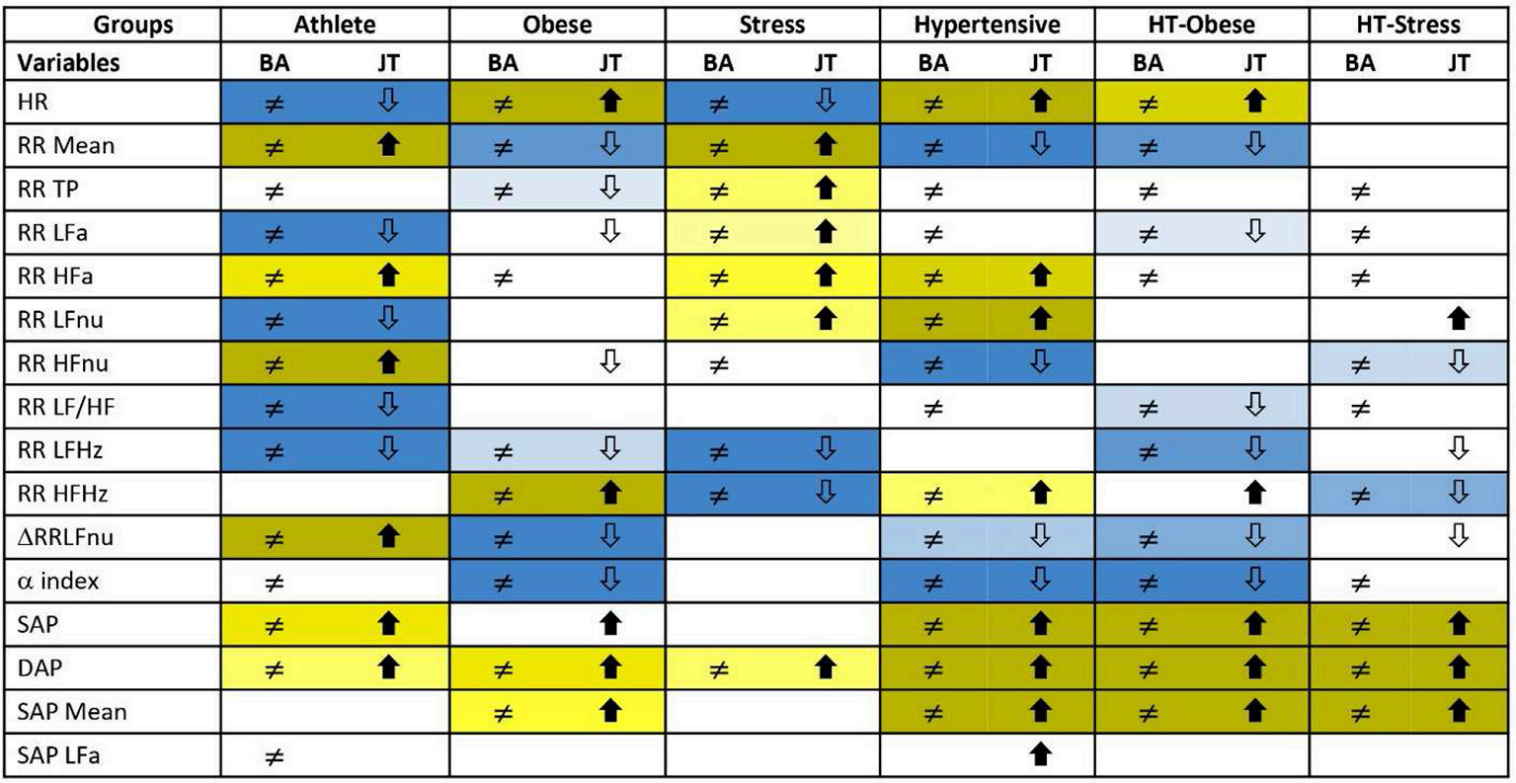
Significance diagram summing up the results of BA and JT permutation tests applied to the adjusted ANS proxies within each test group. Normal group is the reference term in all the comparisons. Columns with header BA: Two-sided BA permutation test (at 0.05 level) in the comparison between the population density functions *f*_*g*_(*x*) of test group *g* and *f*_*N*_(*x*) of normal group, resp.: ≠ stands for significantly different population density functions for at least one *x*, a blank cell denotes equal density curves (Figure 1, step 1). Columns with header JT: One-sided JT test (at 0.05 level) in the comparison between the two population distribution functions *F*_*g*_(*x*) of test group *g* and *F*_*N*_(*x*) of normal group: 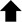 denotes *F*_*g*_(*x*) significantly higher than *F*_*N*_(*x*) for at least one *x*; 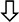 denotes *F*_*g*_(*x*) significantly lower than *F*_*N*_(*x*) for at least one *x*. A blank cell denotes a non-significant comparison (Figure 1, step 1). Background color shades for joint significance of BA and JT tests: - JT test with significant alternative 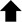 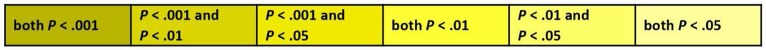 - JT test with significant alternative 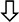 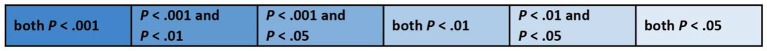
.

Figure 3 reports the significance diagram concerning the Adj-ANS proxies resulted individually discriminant in each “test-vs.-reference” comparison according to the BA and JT tests jointly considered. Inequality symbol denotes BA significant results. Solid up- and empty down-arrows mark JT significant results (i.e., Adj-ANS proxy values greater/smaller than the normal group, respectively). We regard an Adj-ANS proxy as individually discriminant in a “test-vs.-reference” comparison if both BA and JT test results are significant. In this way, the probability of the overall type I error concerning the null hypothesis of no difference in a “test-vs.-reference” comparison of each Adj-ANS proxy is reduced to (0.05)^2^ = 0.0025. That is in line with a more conservative approach that makes rejecting the null hypothesis in favor of the alternative of individual discrimination more difficult. A proxy of the level of strength in individual discrimination capability is given here by the magnitude of the BA and JT *p*-values jointly considered and is indicated in the diagram by cells with different background color shades (the darkest/lightest shade denotes the strongest/less strong level of joint significance). On the other hand, blank cells stand for at least a non-significant result and thus denote the absence of individual discrimination capability.

By the significance diagram, it is apparent that, controlling for age and gender effects, athletes tend to have higher values of RR Mean, RR HFa, RR HFnu, ΔRRLFnu, SAP, and DAP than normal individuals (solid up-arrow), and lower values of HR, RR LFa, RR LFnu, RR LF/HF, and RR LFHz (empty down-arrow). Moreover, the most individually discriminant ANS proxies turn out to be RR Mean, RR HFnu, ΔRRLFnu (the darkest yellow cells), and HR, RR LFa, RR LFnu, RR LF/HF, and RR LFHz (the darkest blue cells). On the other hand, hypertensive individuals tend to have higher values of HR, RR HFa, RR LFnu, RR HFHz, SAP, DAP, and SAP Mean (solid up-arrow), and lower values of RR Mean, RR HFnu, ΔRRLFnu, and α index (empty down-arrow), while the most individually discriminant ANS proxies are HR, RR LFnu, SAP, DAP, SAP Mean (the darkest yellow cells), and RR Mean, RR HFnu, and α index (the darkest blue cells).

Regarding setting-up of ANS differentiation profiles (step 2, Figure 1), the main results of factor analysis carried out for extracting ANS latent domains are given in Table 4. Total communality amounts here to 76.67% of the total variance. In line with the above definition, ANS latent domains are given by the first four common factors, which together account for 86.9% of total communality (Table 4). Each factor explains more than 10% of total communality. Specifically, the first factor (40.84% of total communality) represents the Oscillatory Domain (all indices of rhythms are in normalized units), being highly positively correlated with RR HFnu and ΔRRLFnu, and negatively correlated with RR LF/HF and RR LFnu (Table 4, first column). The second factor (18.04% of total communality) is the Amplitude Domain because of its highly positive correlations with RR TP, RR HFa, RR LFa, and α index (all indices in raw values, Table 4, second column). The third factor (16.48% of total communality) is the Pressure Domain, being highly positively correlated with SAP, DAP, and SAP Mean (Table 4, third column). The fourth factor (11.58% of total communality) represents the Pulse (rate) Domain for its highly positive correlation with HR and negative correlation with RR Mean (Table 4, fourth column). Three of the ANS proxies (i.e., RR HFHz, RR LFHz, and SAP LFa) prove to be linked far weakly with these four factors and then with the ANS latent domains. Accordingly, they are discarded from the analyses subsequently performed to detect discriminant ANS domains (Figure 1, step 2).

**Table 4:** Detection of ANS latent domains by factor analysis: Rotated factor pattern matrix with the varimax method arrested to the first four factors.

**Variables**	**Factor1**	**Factor2**	**Factor3**	**Factor4**
RR HFnu	92*	10	−12	−14
ΔRRLFnu	65*	12	−22	−8
RR LF/HF	−57*	−2	6	11
RR LFnu	−96*	−7	8	11
RR TP	15	96*	−11	−17
RR HFa	40	77*	−11	−9
RR LFa	-21	74*	−11	−15
α index	17	63*	−27	−25
SAP	−12	−12	96*	3
SAP Mean	−11	−13	91*	3
DAP	−19	−14	82*	8
HR	−15	−20	9	96*
RR HFHz	−14	−9	−3	29
RR Mean	13	19	−7	−94*
RR LFHz	12	6	−8	17
SAP LFa	−14	5	12	3
% of total communality	40.84%	18.04%	16.48%	11.58%
cumulative % of total communality	40.84%	58.88%	75.36%	86.94%

*Total communality (i.e., total reproduced variance) = 12.267, total variance = 16, percentage of total variance explained = 76.67%. Printed values are correlation coefficients multiplied by 100 and rounded to the nearest integer. Asterisks flag correlation coefficients greater than, or equal to, 0.4 in absolute value. Interpretation of the first four factors: Factor 1 = Oscillatory domain (variables colored in lilac), Factor 2 = Amplitude domain (variables in pink), Factor 3 = Pressure domain (variables in green), Factor 4 = Pulse domain (variables in blue)*.

**Figure 4:**
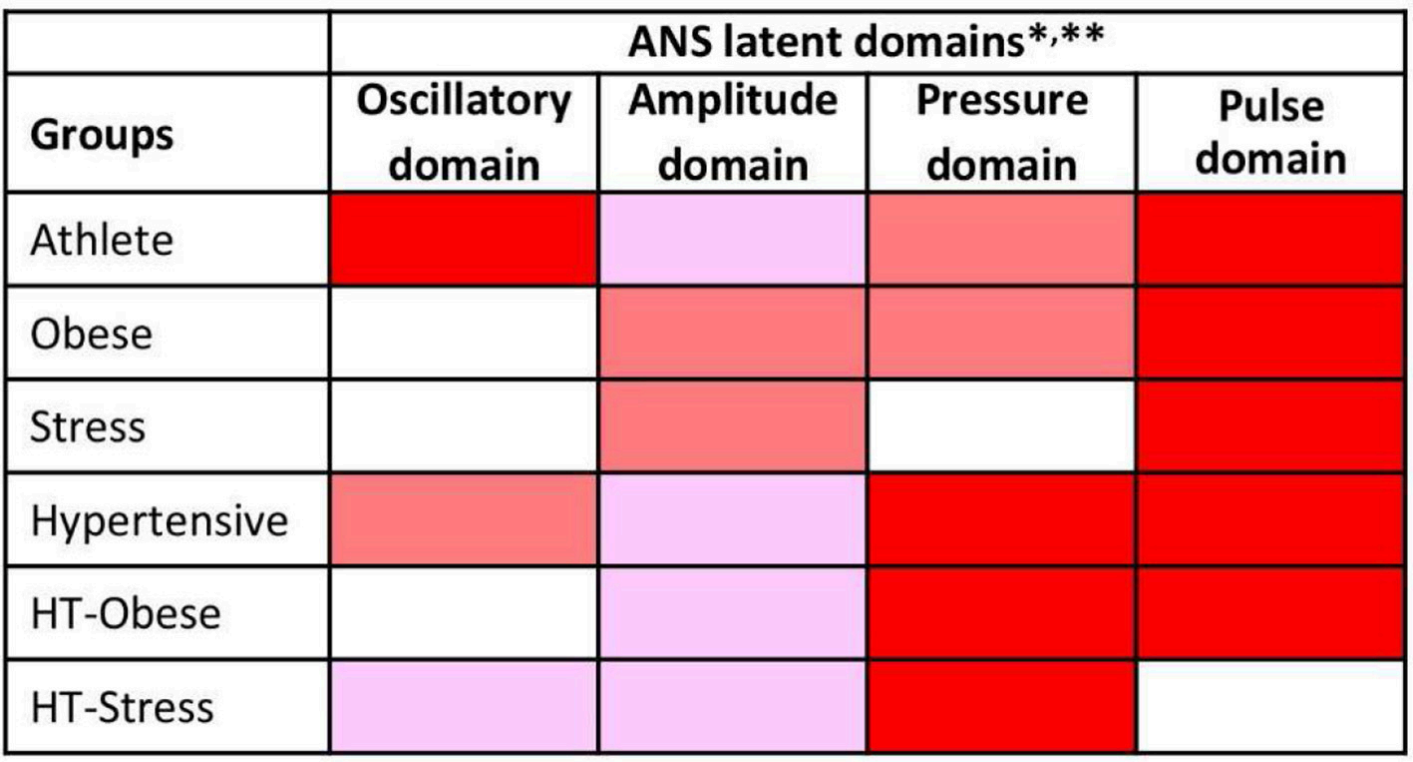
ANS differentiation map of the test groups toward the normal group. *ANS differentiation profiles are given for each test group by the concurrence of the discriminant ANS domains depicted in red, dark pink and light pink according to their strength of discrimination capability toward the normal group. 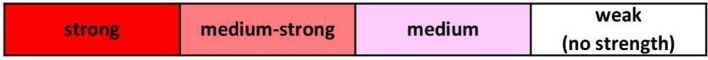 This strength is assessed as the number of jointly significant results on BA and JT tests occurred for the Adj-ANS proxies connected with each specific ANS latent domain. Red cells denote the strongest level of discrimination, where all the tests involving Adj-ANS proxies in that domain are significant. Dark pink cells indicate a medium-strong level of discrimination, in which there is at least a majority of significant results on both BA and JT tests. Light pink cells denote a medium level of discrimination, where there is at least a majority of significant results on either BA or JT tests. Finally, white cells indicate that there are not enough significant test results to regard a specific ANS latent domain as discriminant toward the normal group. **As a result of factor analysis (Table 4), ANS latent domains are connected with the following ANS proxies: Oscillatory domain = RR LFnu, RR HFnu, RR LF/HF, and ΔRRLFnu; Amplitude domain = RR TP, RR LFa, RR HFa, and α index; Pressure domain = SAP, DAP, and SAP Mean; Pulse domain = HR and RR Mean.

Finally, Figure 4 reports the ANS differentiation map, i.e., the graphical map of the ANS differentiation profiles set up for every test group as described in Figure 1, step 2. Colored cells represent the discriminant ANS domains, and are shaded differently according to their level of discrimination capability (Figure 1, step 2). For example, athletes' ANS differentiation profile consists of all the four domains together. Oscillatory and pulse domains have the strongest discrimination capability, pressure domain has a medium-strong level, and amplitude domain a medium level. Moreover, by the significance diagram (Figure 3) it can be seen that inside the oscillatory domain, controlling for age and gender effects, values of RR HFnu and ΔRRLFnu tend to be higher than in normal individuals, while RR LFnu and RR LF/HF tend to have lower values. Similarly, inside the pulse domain, HR is lower, and RR Mean is higher than in normal individuals (Figure 3). In pressure domain, SAP and DAP are higher, while in amplitude domain RR LFa is lower and RR HFa is higher than in normal individuals. ANS differentiation profile of hypertensive subjects is also formed by the four ANS domains together (pressure and pulse domains with the strongest discrimination capability). Obese group differs from the normal group for pulse (strongest discrimination), pressure and amplitude domains, similarly to HT-Obese group (pulse and pressure domains with the strongest discrimination capability). Pulse (strongest discrimination) and amplitude domains characterize Stress group, while pressure (strongest discrimination), oscillatory, and amplitude domains constitute the ANS differentiation profile of HT-Stress.

## Discussion

This investigation on a relatively large population of ambulant subjects shows that multiple statistical tools used in an integrated way can be profitably applied to the analysis of cardiovascular variability. In particular, it suggests that clustering of ANS proxies according to hidden factors (Thompson, 2004; Lucini et al., 2014) might help differentiate properties of clinical groups (athletes, obese subjects, people with high stress, and hypertensives) from controls. To this end, we employ a three-step statistical analysis. We use 2-way ANOVA residuals instead of raw values of the ANS proxies to account for age and gender effects. We employ non-parametric statistical procedures to identify discriminant Adj-ANS proxies. Finally, we set up ANS differentiation profiles by detecting which ANS latent domains have the highest capacity to discriminate HRV properties of clinical groups vs. controls.

### Statistics: a novel tool to interpret autonomic proxies

Usual studies on autonomic innervation representing ANS proxies as raw values must deal with several potential confounders. First, autonomic proxies show an important age (Jandackova et al., 2016) and gender (Dart et al., 2002) dependency, which might hinder clinical applications and affect the capacity to discriminate between clinical groups.

In the present study, we have avoided this possible bias by removing age and gender effects from the considered ANS proxies and obtaining so-called *adjusted ANS proxies* (Adj-ANS proxies), (Figure 1, preliminary step). This step permits to assess the discrimination capability (clinical groups against controls) of ANS proxies and ANS latent domains free of age and gender effects. Accordingly, this approach avoids the drawback of the difficulty of stratifying the subjects within the clinical groups in age and gender classes of adequate size and composition, as already discussed in the Statistics section. In this respect, we argue that resorting to a *de facto* statistical remedy, i.e., the adjustment of the ANS proxies using a statistical model (2-way ANOVA), has the advantage of being applicable in every context where stratification of subjects according to auxiliary characteristics (e.g., age and gender) is not feasible.

Moreover, we are still facing the problem of the redundancy of the measures. In other words, we do not know whether all the individually discriminant ANS proxies carry the same discriminant value (Malliani et al., 1997; Lucini et al., 2014), or which one would be better to employ in practice. In this regard, the significance diagram (Figure 3) highlights, with different color shades of the cells, the ANS proxies that result in significant comparisons between clinical groups and control. It should be noticed that these shades represent the empirical significance levels (i.e., the *p*-values) of BA and JT tests jointly considered and not the *pure* discriminant power of the single ANS proxies.

Factor analysis is a statistical tool that helps unravel hidden links between variables (Thompson, 2004). It also provides clusters of variables that carry homogeneous overall meaning. It appears particularly valuable in this context since it permits to formulate hypotheses about the type of information carried by the extracted clusters of ANS proxies. In doing so, it combines statistics with underlying neural physiology. Here we have shown that the information underlying the considered 16 ANS proxies can be represented with a very good degree of approximation by four common factors (whose fraction of information is of sufficient amplitude: at least 10%). The combination of variables strongly linked to the four hidden factors may suggest an underlying meaning and physiological interpretation. More in detail, near 87% of the total communality (Table 4) is explained by the first four latent factors. They refer to: (1) oscillatory behavior (oscillatory domain, in nu) (Gerstner et al., 1997; Buzsáki and Watson, 2012); (2) total variance, oscillatory raw values and alpha index (amplitude domain, in absolute units) (La Rovere et al., 1998; Pagani and Malliani, 2000); (3) raw values of arterial pressure (pressure domain), and (4) raw values of heart rate and RR interval (pulse domain). It seems therefore that the major part of information carried by ANS proxies could provide a window on the two principal coding of cardiovascular variability (oscillations and amplitude, i.e., first and second factor) (Pagani and Malliani, 2000) and simple hemodynamic measures (arterial pressure and pulse rate, i.e., third and fourth factor). Importantly this approach might help resolve (at least as a first approximation) the riddle of which autonomic indices should be clinically employed. In fact, it provides information on how hidden factors *govern* major aspects of cardiovascular variability. As a corollary, since all information about HRV and arterial pressure findings can be summarized in four uncorrelated factors, we may propose that this approach could be employed to describe and monitor autonomic regulation and its changes during relevant conditions. Just as an example, we may consider managing training season in athletes, or monitor the effects of stress and recovery, or of diets interventions in obese individuals.

In this context, the previous report of a strong coherence between RR and ANS rhythms, according to the “concept of common central mechanisms governing sympathetic and parasympathetic rhythmic activity” (Pagani et al., 1997), seem to imply a greater strength of oscillatory (i.e., nu) than amplitude (i.e., raw values) information.

### Individual discrimination vs. joint discrimination, discrimination capability vs. discrimination power

As already mentioned, a fundamental step in the study was to assess the individual discrimination capability of the Adj-ANS proxies in the comparison between test groups and the reference normal group in order to define the ANS differentiation profiles. On this point, two remarks are worth making. First, we have decided to proceed variable-by-variable and assess what we have denoted as individual discrimination capability. In this way, we intended to give some practical indications directly usable in clinical terms about which ANS proxies could help recognize subjects that are outside the normal group. As an alternative, we could have proceeded by considering the ANS proxies *jointly* and detecting the most discriminant ones *each net of the others*. This would have required us to work within a genuine statistical modeling approach, by specifying a suitable functional form linking the variables along with formulating a set of conjectures about the distribution of the data. Nevertheless, this was outside our objective. At this stage of our exploration, we feel that forcing the data in order that they meet relationships still under investigation may be too premature. As another alternative, we could have tested the discrimination capability of the ANS latent domains produced by factor analysis, rather than the single ANS proxies, thus working indirectly on the clusters of the ANS proxies connected each to a specific HRV domain. However, this approach has two main drawbacks. The common factors representing the ANS latent domains are not observable variables so that any analysis concerning their individual discrimination capability would lead to indications not directly usable in clinical practice. Moreover, the common factors are extracted by the principal factor method, and then from the part of the multidimensional variability shared by all the ANS proxies. Consequently, the specificity of each ANS proxy, which instead is the part of the variability not in common, would have been *de facto* excluded from the discrimination capability analysis.

Accordingly, at this stage, we have chosen to apply the non-parametric BA and JT testing procedures to each single ANS proxy (adjusted for age and gender effects) in order to: (1) carry out “test-vs.-reference” comparisons variable-by-variable without introducing a priori assumptions on the ANS proxy distributions (most ANS proxies, both in original and adjusted units, are not normally distributed), (2) perform the above comparisons by considering the distributions of the ANS proxies in their entirety, (3) draw conclusions about the individual discrimination capability of each ANS proxy using two different statistical testing procedures, to obtain as more robust results as possible.

Second, strictly related to the point above there is the implicit distinction, which ultimately affected the choice of the statistical approach, between discrimination capability and discrimination power. All the analyses we performed were addressed to detecting the potential capability of the ANS proxies of distinguishing a test group from the normal one in a wider population of subjects. As already pointed out, this is not the same as evaluating the discrimination power of the ANS proxies because, in general, it is one thing to assess if a relation exists, quite another to say how strong that relation is. Then, since we are at an exploratory stage of the investigation, we have preferred to focus here on the existence of the discrimination capability of each ANS proxy, and defer any inspection toward their discrimination power in future studies.

### The differentiation profile

Traditionally comparison among groups is provided by the difference from controls of various paradigmatic groups. Athletes show, e.g., what could be interpreted as a vagal shift (Iellamo et al., 2002; lower Heart Rate, greater RR variance, smaller LF in nu, higher alpha index), combined with the greatest value of increase LF nu with standing up (suggestive of greatest sympathetic responsiveness, a much valued element in competitive sport; Manzi et al., 2009).

From a practical point of view, we remind that a similar profile can be observed in the distribution of raw and adjusted values. A detailed statistical comparison with BA and JT non-parametric tests suggests that differences between individual paradigmatic groups and controls may be condition specific (Jänig, 2008; Table 3 and Figures 2, 3). Moreover, BA and JT tests may disclose subtle nuances of hidden coding modalities (Gerstner et al., 1997; Buzsáki and Watson, 2012) between different indices that might merit a deeper inquiry, as furnished by factor analysis (Thompson, 2004; Table 4) according to a unique approach. In brief, with BA and JT we perform comparisons between the distributions of individual ANS proxies in clinical groups and controls, while with factor analysis we reduce the number of meaningful proxies to only four, and hence we can largely simplify the assessment of the main traits distinguishing clinical groups from controls (Figure 4). Instead of dealing with the small portion of information individually distributed across all autonomic indices, we can rely on the strength of the ANS differentiation profiles, i.e., the discriminant ANS latent domains disclosed by combining factor analysis with the test-vs.-reference comparisons based on BA and JT tests. Notably, this approach, which simultaneously uses all available information about the system (Haken, 1977), shows that e.g., athletes (Figure 4) differ from controls in all domains, but with a graded strength: maximal in the (normalized) oscillatory and in the pulse domains, slightly less in the pressure domain, and minimal in the amplitude domain.

Also, the hypertensive group differs (Lucini et al., 2014) from control in all the four domains, but with a different grading (maximal strength for pressure and pulse domains, less so for amplitude and oscillatory domains). Obese (Peterson et al., 1988), Ht-Obese and Ht-Stress groups differ in three differentiating domains. The Stress group (Lucini et al., 2002) differs in only two domains (amplitude and pulse). We posit in addition that for clinical applications the ANS differentiation map (as in Figure 4) permits to rapidly and simply synthesize the possible difference between clinical groups and controls, evidencing the ANS latent domains that have at least a medium strength of discrimination. While the ANS differentiation map considers, in practice, clusters of ANS proxies, the significance diagram (Figure 3) permits to identify the single ANS proxies inside each ANS latent domain that resulted in significant comparisons according to BA and JT tests. This aspect may also help define ANS investigations addressing differences between clinical groups on a more rational basis.

Lastly, it is worth pointing out that the statistical methodology we used here for setting up ANS differentiation profiles is broader in scope. It is given by the integrated use of statistical analysis methods that, from a theoretical point of view, were developed irrespective of specific fields of application. Consequently, we argue that, after due adjustments, this methodology could be as well employed to study ANS differences of other non-normal conditions, or even applied to detect differentiation profiles among groups in other completely different contexts.

### Limitations

Important limitations must be recognized in this pragmatic protocol (Ford and Norrie, 2016). First, this is an indirect study, comparing groups at a one-time point only, but following usual clinical routines. Moreover, the study population is large, and we utilize extensively, we believe for the first time, a set of integrated statistical procedures capable of providing a differentiation map, showing which cluster of variables best indicate the ANS difference between test groups and controls.

In addition, we do not present direct data on activity and syntax of neurovegetative neurons (Buzsáki and Watson, 2012). Indirect is, in particular, the nature of autonomic indices that are employed in this investigation. However, the integrated use of multiple statistical tools permitted to provide empirical support to the hypothesis that autonomic codes are expressed in either amplitude or oscillations (Pagani and Malliani, 2000). Present findings derive from the prevailing use of a data-driven (instead of model-based) approach to the analyses, which allowed us to build the ANS differentiation map directly from the data, without the need for a priori assumptions on the distributions of the ANS proxies and their mutual relationships.

Moreover, given that the composition of the groups was not homogeneous with respect to age and gender, we were forced to rely on a statistical remedy, i.e., performing the analyses on the ANS proxies adjusted for age and gender effects instead of the original ones. Overall, in the “test-vs.-reference” comparisons carried out using the Adj-ANS proxies we have obtained results in line with the literature, e.g., in the hypertensive group results concerning ΔRRLFnu and α index, which are the proxies with the highest informative content for this group, are congruent with what is expected (Lucini et al., 2014). There are, however, few exceptions. Again, in the hypertensive group, net of age and gender effects, RR HFa results significantly greater than in the normal group (Figure 3), while we would have expected the opposite situation (Table 3). This anomaly could be because we have used the adjusted ANS proxies, with respect to which there is no comparability with the literature yet.

In spite of a relatively large overall population, combined conditions (such as stress, obesity, and hypertension) may lead to very small subgroups, rendering impossible to interpret confounding effects, or generalizing all findings. This would require focused studies, possibly with specific interventions with a longitudinal design.

## Conclusions

The application of a pragmatic approach (Ford and Norrie, 2016), designed to show the behavior of ANS proxies close to real life rather than in stringent laboratory conditions, and the prevailing use of data-driven statistical methods on a large dataset of several different paradigmatic groups of ambulant subjects indicate that ANS variables cluster in a small number of latent factors. Each factor is strongly linked to few homogeneous proxies of autonomic modulation. The properties of latent factors might therefore suggest a novel way to interpret underlying physiological mechanisms.

Thus the application of multiple (non-parametric and exploratory) statistical and graphical tools to ANS proxies defines differentiation profiles that could pave the way to a better understanding of autonomic differences between clinical groups and controls, with potential beneficial effects on clinical applications.

## Author contributions

DL, NS, and MP contributed to the study design; DL and MP contributed to drafting the manuscript; NS contributed to statistical analysis; DL, NS, and MP contributed to critically revising the text; DL, NS, and MP contributed to and approved the final version of the text.

### Conflict of interest statement

The authors declare that the research was conducted in the absence of any commercial or financial relationships that could be construed as a potential conflict of interest.
