# Supplementary material for: Autonomic Differentiation Map: A Novel Statistical Tool for Interpretation of Heart Rate Variability
Source: Front Physiol. 2018 Apr 23;9:401. doi: 10.3389/fphys.2018.00401 (PMC5924813; doi:10.3389/fphys.2018.00401)
Supplement: Supplementary file 1 [file Table1.DOCX]

Supplementary Material

**Autonomic differentiation map: a novel statistical tool for interpretation of heart rate variability**

**Daniela Lucini^1^*, Nadia Solaro^2^  and Massimo Pagani^1^***

**Correspondence:** Daniela Lucini, MD, PhD: [daniela.lucini@unimi.it](mailto:daniela.lucini@unimi.it)

# Supplementary Figures and Tables

## Supplementary Figures

**Supplementary Figure S1.** Box plots of within-groups distributions of HR, RR TP, RR LFnu, ΔRRLFnu, α index and SAP

**
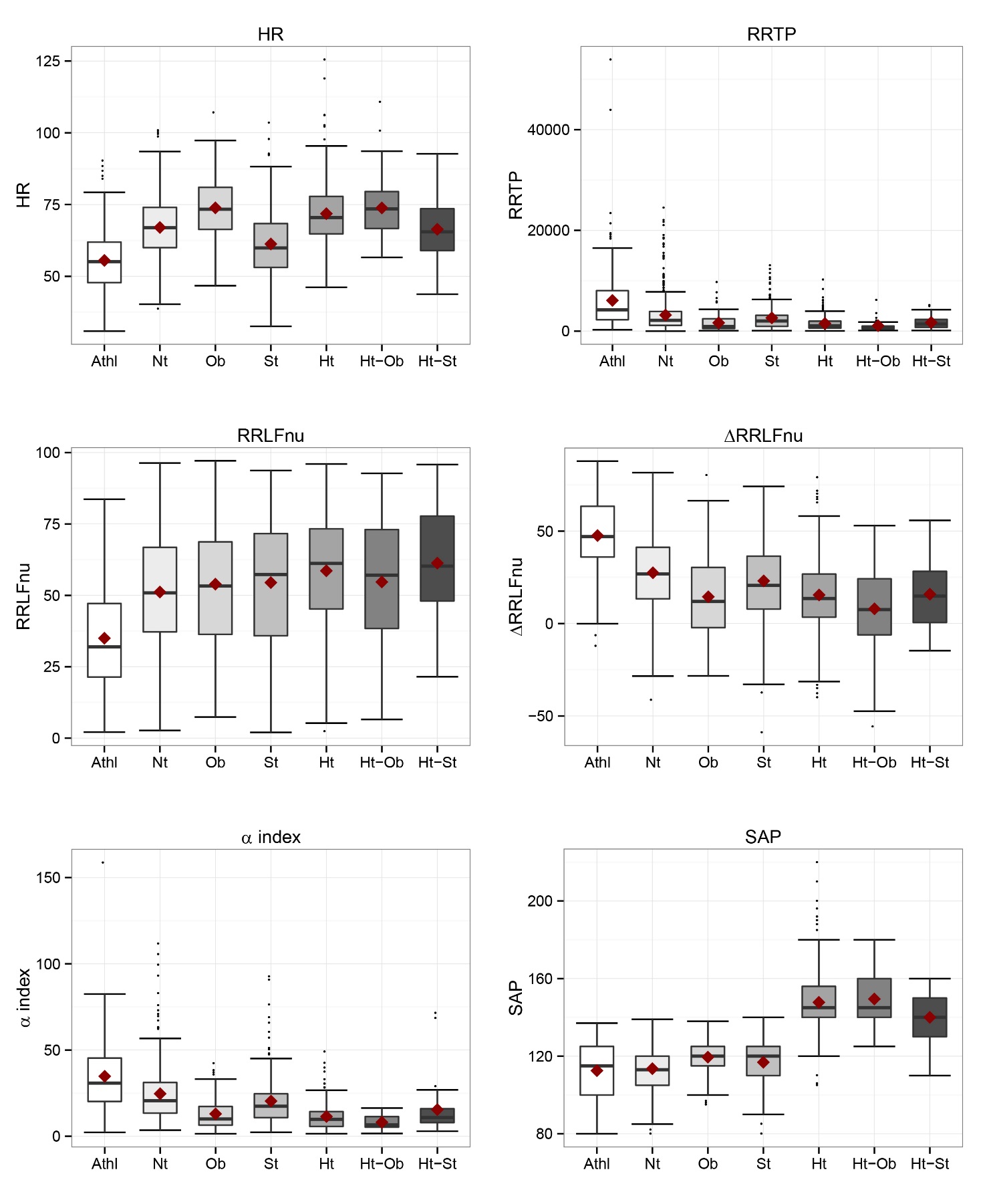
**

*Legend*.

Labels: Athl=Athlete, Nt=Normal, Ob=Obese, St=Stress, Ht=Hypertensive, Ht-Ob=Hypertensive Obese, Ht-St=Hypertensive-Stress.

Red diamonds within each box denote within-group means.

**Supplementary Figure S2.** Box plots of within-groups distributions of adjusted ANS proxies (i.e. ANOVA residuals) of HR, RR TP, RR LFnu, ΔRRLFnu, α index and SAP


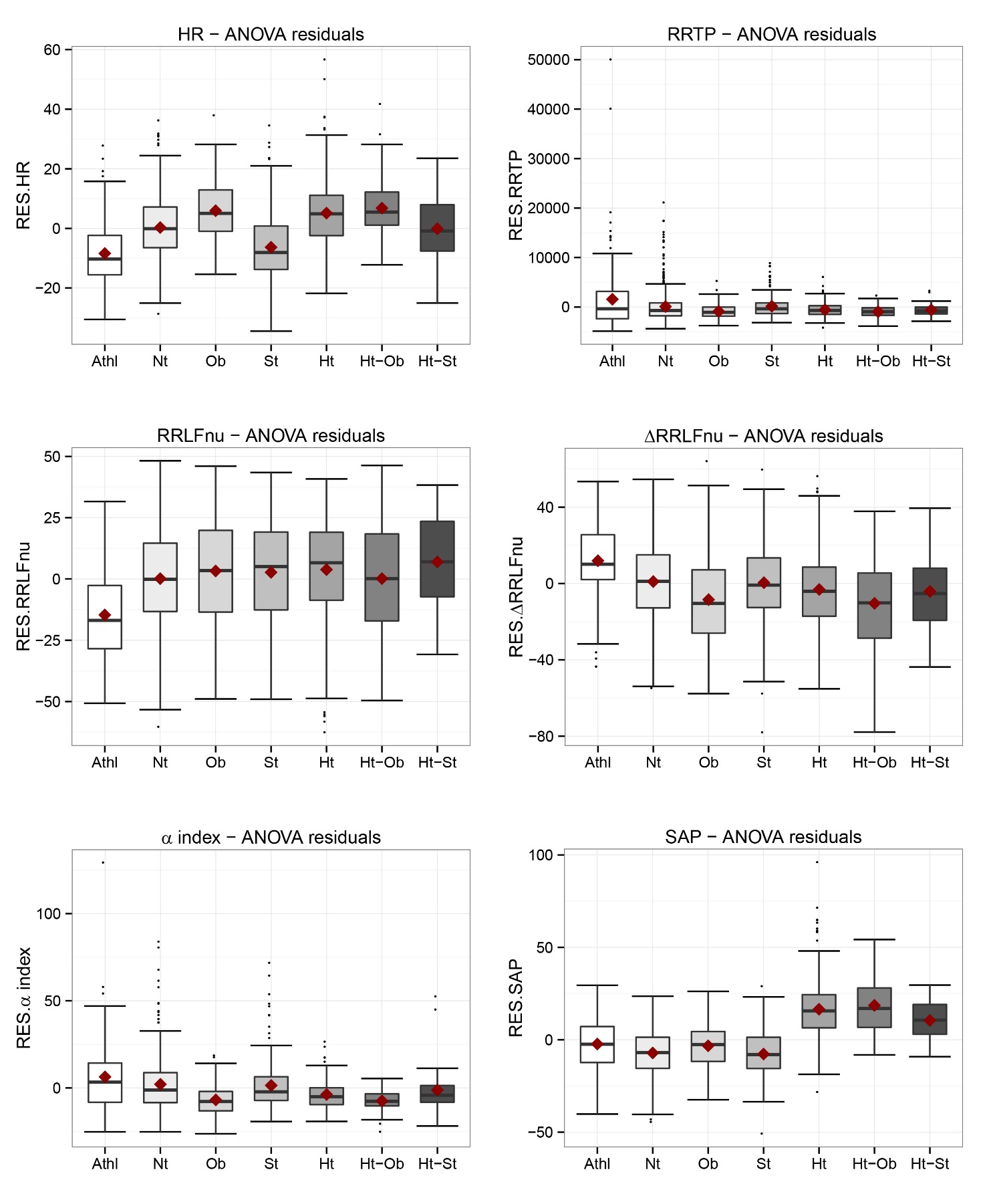


*Legend*. Adjusted ANS proxies are corrected for age and gender effects.

Labels: Athl=Athlete, Nt=Normal, Ob=Obese, St=Stress, Ht=Hypertensive, Ht-Ob=Hypertensive Obese, Ht-St=Hypertensive-Stress.

Red diamonds within each box denote within-group means.

**Supplementary Figure S3.** Number of significant results concerning the BA test for each test group and ANS latent domain


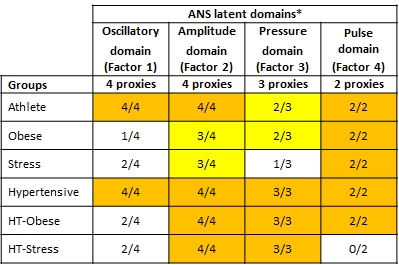


*Legend*.

*As a result of factor analysis (Table 4 and Supplementary Table S6), ANS latent domains are connected with the following ANS proxies: Oscillatory domain = RRLFnu, RR HFnu, RR LF/HF and ΔRRLFnu; Amplitude domain = RR TP, RR LFa, RR HFa, α index; Pressure domain = SAP, DAP and SAP Mean; Pulse domain = HR and RR Mean.

Cells in the table are colored differently according to the number of significant results on the BA test (Supplementary Table S5, Part A, and Figures 2 and 3 in the text). Orange cells denote the situation in which all the results concerning the Adj-ANS proxies in that domain are significant at 0.05 level. Yellow cells indicate that significance is obtained only for a majority of the Adj-ANS proxies in that domain. White cells denote the situation in which there are not enough significant test results.

**Supplementary Figure S4.** Number of significant results concerning the JT test for each test group and ANS latent domain


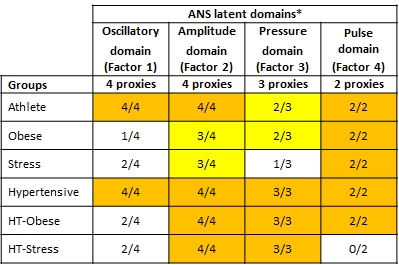


*Legend*.

*As a result of factor analysis (Table 4 and Supplementary Table S6), ANS latent domains are connected with the following ANS proxies: Oscillatory domain = RRLFnu, RR HFnu, RR LF/HF and ΔRRLFnu; Amplitude domain = RR TP, RR LFa, RR HFa, α index; Pressure domain = SAP, DAP and SAP Mean; Pulse domain = HR and RR Mean.

Cells in the table are colored differently according to the number of significant results on the JT test (Supplementary Table S5, Part B, and Figure 3 in the text). Orange cells denote the situation in which all the results concerning the Adj-ANS proxies in that domain are significant at 0.05 level. Yellow cells indicate that significance is obtained only for a majority of the Adj-ANS proxies in that domain. White cells denote the situation in which there are not enough significant test results.

## Supplementary Tables

**Supplementary Table S1.** Distribution of individuals by gender within clinical groups

| **Gender** | | **Groups** | | | | | | | **Total** |
| --- | --- | --- | --- | --- | --- | --- | --- | --- | --- |
|  |  | **Athlete** | **Normal** | **Obese** | **Stress** | **Hypertensive** | **HT-Obese** | **HT-Stress** |  |
| Female | *count* | 43 | 303 | 77 | 123 | 103 | 25 | 15 | 689 |
|  | *%* | 28.9% | 56.5% | 75.5% | 64.7% | 39.0% | 45.5% | 39.5% | 51,6% |
| Male | *count* | 106 | 233 | 25 | 67 | 161 | 30 | 23 | 645 |
|  | *%* | 71.1% | 43.5% | 24.5% | 35.3% | 61.0% | 54.5% | 60.5% | 48,4% |
| **Total** | *count* | 149 | 536 | 102 | 190 | 264 | 55 | 38 | 1334 |
|  | *%* | 100.0% | 100.0% | 100.0% | 100.0% | 100.0% | 100.0% | 100.0% | 100.0% |

**Supplementary Table S2.** Distribution of individuals by classes of age within clinical groups

| **Age in class** | | **Groups** | | | | | | | **Total** |
| --- | --- | --- | --- | --- | --- | --- | --- | --- | --- |
|  |  | **Athlete** | **Normal** | **Obese** | **Stress** | **Hypertensive** | **HT-Obese** | **HT-Stress** |  |
| ≤ 34 | *count* | 146 | 278 | 31 | 55 | 37 | 9 | 5 | 561 |
|  | *%* | 98.0% | 50.8% | 31.3% | 28.9% | 13.7% | 16.4% | 13.2% | 41.6% |
| 35 – 49 | *count* | 3 | 172 | 41 | 81 | 104 | 21 | 18 | 440 |
|  | *%* | 2.0% | 31.4% | 41.4% | 42.6% | 38.4% | 38.2% | 47.4% | 32.6% |
| ≥ 50 | *count* | 0 | 97 | 27 | 54 | 130 | 25 | 15 | 348 |
|  | *%* | 0.0% | 17.7% | 27.3% | 28.4% | 48.0% | 45.5% | 39.5% | 25.8% |
| **Total** | *count* | 149 | 547 | 99 | 190 | 271 | 55 | 38 | 1349 |
|  | *%* | 100.0% | 100.0% | 100.0% | 100.0% | 100.0% | 100.0% | 100.0% | 100.0% |

**Supplementary Table S3.** Distribution of individuals by gender and classes of age within clinical groups

| **Gender** | | | | **Groups** | | | | | | | **Total** |
| --- | --- | --- | --- | --- | --- | --- | --- | --- | --- | --- | --- |
|  |  |  |  | **Athlete** | **Normal** | **Obese** | **Stress** | **Hypertensive** | **HT-Obese** | **HT-Stress** |  |
| Female | **Age in class** | ≤ 34 | *count* | 42 | 160 | 25 | 42 | 12 | 4 | 2 | 287 |
|  |  |  | *%* | 97.7% | 52.8% | 33.8% | 34.1% | 11.7% | 16.0% | 13.3% | 41.8% |
|  |  | 35 – 49 | *count* | 1 | 96 | 33 | 49 | 32 | 8 | 5 | 224 |
|  |  |  | *%* | 2.3% | 31.7% | 44.6% | 39.8% | 31.1% | 32.0% | 33.3% | 32.7% |
|  |  | ≥ 50 | *count* | 0 | 47 | 16 | 32 | 59 | 13 | 8 | 175 |
|  |  |  | *%* | 0.0% | 15.5% | 21.6% | 26.0% | 57.3% | 52.0% | 53.3% | 25.5% |
|  | **Total** | | *count* | 43 | 303 | 74 | 123 | 103 | 25 | 15 | 686 |
|  |  |  | *%* | 100.0% | 100.0% | 100.0% | 100.0% | 100.0% | 100.0% | 100.0% | 100.0% |
| Male | **Age in class** | ≤ 34 | *count* | 104 | 118 | 6 | 13 | 25 | 5 | 3 | 274 |
|  |  |  | *%* | 98.1% | 50.6% | 24.0% | 19.4% | 15.5% | 16.7% | 13.0% | 42.5% |
|  |  | 35 – 49 | *count* | 2 | 76 | 8 | 32 | 72 | 13 | 13 | 216 |
|  |  |  | *%* | 1.9% | 32.6% | 32.0% | 47.8% | 44.7% | 43.3% | 56.5% | 33.5% |
|  |  | ≥ 50 | *count* | 0 | 39 | 11 | 22 | 64 | 12 | 7 | 155 |
|  |  |  | *%* | 0.0% | 16.7% | 44.0% | 32.8% | 39.8% | 40.0% | 30.4% | 24.0% |
|  | **Total** | | *count* | 106 | 233 | 25 | 67 | 161 | 30 | 23 | 645 |
|  |  |  | *%* | 100.0% | 100.0% | 100.0% | 100.0% | 100.0% | 100.0% | 100.0% | 100.0% |

**Supplementary Table S4.** F test *p*-values of the 2-way full ANOVA model explaining each ANS proxy with age and gender main effects and interaction

| **ANS proxies** | **Age main effect** | **Gender main effect** | **Age-by-gender**  **interaction** |
| --- | --- | --- | --- |
| HR | .000^†††^ | .003^††^ | .000^†††^ |
| RR Mean | .001^†††^ | .000^†††^ | .000^†††^ |
| RR TP | .000^†††^ | .129 | .619 |
| RR LFa | .000^†††^ | .066 | .465 |
| RR HFa | .000^†††^ | .635 | .732 |
| RR LFnu | .000^†††^ | .309 | .214 |
| RR HFnu | .000^†††^ | .747 | .144 |
| RR LF/HF | .000^†††^ | .826 | .127 |
| RR LFHz | .000^†††^ | .759 | .721 |
| RR HFHz | .075 | .800 | .233 |
| ΔRRLFnu | .000^†††^ | .129 | .072 |
| α index | .000^†††^ | .000^†††^ | .003^††^ |
| SAP | .000^†††^ | .000^†††^ | .000^†††^ |
| DAP | .000^†††^ | .002^††^ | .605 |
| SAP Mean | .000^†††^ | .000^†††^ | .001^†††^ |
| SAP LFa | .572 | .001^†††^ | .020^†^ |

Note: Significance codes: .001^†††^, .01^††^, .05^†^

**Supplementary Table S5.** *P*-values of two-sided Bowman-Azzalini’s (BA) test and one-sided Jonckheere-Terpstra’s (JT) test for both increasing and decreasing alternatives in the comparison of Athlete, Obese, Stress, Hypertensive (HT), HT-Obese, HT-Stress groups with Normal group (reference group). Used variables are adjusted ANS proxies*

| ***Part A – Bowman-Azzalini’s permutation test***** | | | | | | | | | | | | |
| --- | --- | --- | --- | --- | --- | --- | --- | --- | --- | --- | --- | --- |
| **Variables** | **Athlete** | | **Obese** | | **Stress** | | **Hypertensive** | | **HT-Obese** | | **HT-Stress** | |
| HR | .000^†††^ | | .000^†††^ | | .000^†††^ | | .000^†††^ | | .009^††^ | | .533 | |
| RR Mean | .000^†††^ | | .002^††^ | | .000^†††^ | | .000^†††^ | | .009^††^ | | .473 | |
| RR TP | .000^†††^ | | .041^†^ | | .009^††^ | | .000^†††^ | | .007^††^ | | .042^†^ | |
| RR LFa | .000^†††^ | | .109 | | .040^†^ | | .000^†††^ | | .047^†^ | | .042^†^ | |
| RR HFa | .000^†††^ | | .048^†^ | | .002^††^ | | .000^†††^ | | .001^†††^ | | .018^†^ | |
| RR LFnu | .000^†††^ | | .191 | | .010^††^ | | .000^†††^ | | .531 | | .205 | |
| RR HFnu | .000^†††^ | | .104 | | .027^†^ | | .000^†††^ | | .698 | | .019^†^ | |
| RR LF/HF | .000^†††^ | | .131 | | .377 | | .000^†††^ | | .006^††^ | | .016^†^ | |
| RR LFHz | .000^†††^ | | .002^††^ | | .000^†††^ | | .085 | | .001^†††^ | | .070 | |
| RR HFHz | .856 | | .000^†††^ | | .000^†††^ | | .002^††^ | | .091 | | .035^†^ | |
| ΔRRLFnu | .000^†††^ | | .001^†††^ | | .116 | | .004^††^ | | .019^†^ | | .324 | |
| α index | .023^†^ | | .000^†††^ | | .054 | | .000^†††^ | | .000^†††^ | | .020^†^ | |
| SAP | .018^†^ | | .070 | | .808 | | .000^†††^ | | .000^†††^ | | .000^†††^ | |
| DAP | .025^†^ | | .012^†^ | | .006^††^ | | .000^†††^ | | .000^†††^ | | .000^†††^ | |
| SAP Mean | .898 | | .002^††^ | | .424 | | .000^†††^ | | .000^†††^ | | .000^†††^ | |
| SAP LFa | .036^†^ | | .597 | | .162 | | .068 | | .053 | | .124 | |
|  | | | | | | | | | | | | |
| ***Part B – One-sided JT permutation test for both increasing (inc.) and decreasing (dec.) alternatives****** | | | | | | | | | | | | |
| **Variables** | **Athlete** | | **Obese** | | **Stress** | | **Hypertensive** | | **HT-Obese** | | **HT-Stress** | |
|  | ***inc.*** | ***dec.*** | ***inc.*** | ***dec.*** | ***inc.*** | ***dec.*** | ***inc.*** | ***dec.*** | ***inc.*** | ***dec.*** | ***inc.*** | ***dec.*** |
| HR | 0^†††^ | 1 | 1 | 0^†††^ | 0^†††^ | 1 | 1 | 0^†††^ | 1 | 0^†††^ | .461 | .542 |
| RR Mean | 1 | 0^†††^ | 0^†††^ | 1 | 1 | 0^†††^ | 0^†††^ | 1 | 0^†††^ | 1 | .525 | .465 |
| RR TP | .775 | .237 | .017^†^ | .980 | .988 | .011^†^ | .329 | .662 | .054 | .945 | .374 | .625 |
| RR LFa | 0^†††^ | 1 | .038^†^ | .959 | .956 | .040^†^ | .199 | .795 | .039^†^ | .957 | .270 | .726 |
| RR HFa | .962 | .040^†^ | .301 | .688 | .992 | .007^††^ | .990 | .009^††^ | .816 | .194 | .590 | .434 |
| RR LFnu | 0^†††^ | 1 | .896 | .105 | .955 | .045^†^ | .999 | .001^†††^ | .553 | .465 | .978 | .022^†^ |
| RR HFnu | 1 | 0^†††^ | .040^†^ | .959 | .063 | .933 | 0^†††^ | 1 | .445 | .567 | .004^††^ | .994 |
| RR LF/HF | 0^†††^ | 1 | .936 | .056 | .808 | .186 | .592 | .425 | .042^†^ | .952 | .791 | .210 |
| RR LFHz | 0^†††^ | 1 | .016^†^ | .989 | 0^†††^ | 1 | .256 | .736 | .002^††^ | .997 | .001^†††^ | .998 |
| RR HFHz | .595 | .392 | 1 | 0^†††^ | 0^†††^ | 1 | 1 | 0^†††^ | .999 | .002^††^ | 0^†††^ | .998 |
| ΔRRLFnu | 1 | 0^†††^ | 0^†††^ | 1 | .261 | .734 | .002^††^ | .998 | .001^†††^ | 1 | .048^†^ | .948 |
| α index | .919 | .070 | 0^†††^ | 1 | .427 | .564 | 0^†††^ | 1 | 0^†††^ | 1 | .087 | .907 |
| SAP | 1 | 0^†††^ | .997 | .004^††^ | .286 | .711 | 1 | 0^†††^ | 1 | 0^†††^ | 1 | 0^†††^ |
| DAP | .995 | .005^††^ | .998 | .001^†††^ | .968 | .025^†^ | 1 | 0^†††^ | 1 | 0^†††^ | 1 | 0^†††^ |
| SAP Mean | .726 | .281 | .993 | .007^††^ | .597 | .399 | 1 | 0^†††^ | 1 | 0^†††^ | 1 | 0^†††^ |
| SAP LFa | .796 | .214 | .913 | .090 | .445 | .548 | .955 | .043^†^ | .677 | .327 | .429 | .574 |

*Notes*: Significance codes: .001^†††^, .01^††^, .05^†^

*Variables considered in the BA and JT tests are adjusted ANS proxies (Adj-ANS proxies), i.e. residuals obtained from the 2-way full ANOVA model including age and gender as explanatory factors through their main effects and interaction and each ANS proxy in turn as dependent variable.

**Null hypothesis in Bowman-Azzalini’s permutation test is: $H_{0} : f_{g}\left( x \right)= f_{N}(x)$ for all $x$, tested against the alternative: $H_{1} : f_{g}\left( x \right)\neq f_{N}(x)$ for any $x$, where $f_{g}(x)$ and $f_{N}(x)$ are the population density functions of ANS proxy $X$ – adjusted for age and gender effects – in groups *g* and *N*, resp., with *N* = normal and *g* = athlete, obese, stress, hypertensive, HT-obese and HT-stress.

***Null hypothesis in Jonckheere-Terpstra’s permutation test is: $H_{0} : F_{g}\left( x \right)= F_{N}(x)$ for all $x$, separately tested against the two alternatives: $H_{1} : F_{g}\left( x \right)\leq F_{N}(x)$ (increasing alternative – inc.) and: $H_{1} : F_{g}\left( x \right)\geq F_{N}(x)$ (decreasing alternative – dec.), resp., with strict inequalities holding for any $x$, where $F_{g}(x)$ and $F_{N}(x)$ are the population distribution functions of ANS proxy $X$ – adjusted for age and gender effects – in groups *g* and *N*, resp. (i.e. $F_{G}\left( x \right)=\Pr_{G} \left( X\leq x \right)$ with $G=g, N$).

**Supplementary Table S6.** Factor analysis with principal factor extraction method: Eigenvalues of the reduced correlation matrix. Total communality (i.e. total reproduced variance) = 12.267, total variance = 16, percentage of total variance explained = 76.67%

| **Factor** | **Eigenvalue** | **Difference** | **Proportion** | **Cumulative** |
| --- | --- | --- | --- | --- |
| 1 | 5.0101 | 2.7971 | 0.4084 | 0.4084 |
| 2 | 2.2129 | 0.1918 | 0.1804 | 0.5888 |
| 3 | 2.0212 | 0.6001 | 0.1648 | 0.7536 |
| 4 | 1.4211 | 0.5421 | 0.1158 | 0.8694 |
| 5 | 0.8790 | 0.3801 | 0.0717 | 0.9411 |
| 6 | 0.4989 | 0.2742 | 0.0407 | 0.9818 |
| 7 | 0.2247 | 0.1476 | 0.0182 | 1.0000 |

*Note:* Prior communality estimates are given by adjusted squared multiple correlations, whose sum is equal to the sum of maximum absolute correlations (Cureton, 1968). During the iterative procedure, communalities estimated greater than 1 were allowed to exceed 1 by means of the option “ultraheywood” implemented in software SAS.

Reference:

Cureton, E. E. (1968), A Factor Analysis of Project TALENT Tests and Four Other Test Batteries, Interim Report 4 to the U.S. Office of Education, Cooperative Research Project No. 3051.) Palo Alto, CA: Project TALENT Office, American Institutes for Research and University of Pittsburgh.
